# Effect of the WeCareAdvisor™ on family caregiver outcomes in dementia: a pilot randomized controlled trial

**DOI:** 10.1186/s12877-018-0801-8

**Published:** 2018-05-10

**Authors:** Helen C. Kales, Laura N. Gitlin, Barbara Stanislawski, H. Myra Kim, Katherine Marx, Molly Turnwald, Claire Chiang, Constantine G. Lyketsos

**Affiliations:** 1 Program for Positive Aging, Department of Psychiatry, University of Michigan, 4250 Plymouth Road, Box 5765, Ann Arbor, MI 48109 USA; 2 Department of Veterans Affairs, HSR&D Center for Clinical Management Research (CCMR), Ann Arbor, MI USA; 3 Geriatric Research, Education and Clinical Center (GRECC), VA Ann Arbor Healthcare System, Ann Arbor, MI USA; 4 Department of Community Public Health, School of Nursing, Johns Hopkins University, Baltimore, MD USA; 5 Division of Geriatrics and Gerontology, School of Medicine, Johns Hopkins University, Baltimore, MD USA; 6 Center for Innovative Care on Aging, Johns Hopkins University, Baltimore, MD USA; 7 Center for Statistical Consultation and Research, University of Michigan, Ann Arbor, USA; 8 Department of Psychiatry and Behavioral Sciences, Johns Hopkins Bayview and Johns Hopkins University, Baltimore, MD USA

**Keywords:** Neuropsychiatric symptoms of dementia, Non-pharmacologic strategies, informal caregivers

## Abstract

**Background:**

Behavioral and psychological symptoms of dementia (BPSD) are universal and associated with multiple negative outcomes. This pilot randomized controlled trial (RCT) evaluated the effect of using the WeCareAdvisor, an innovative web-based tool developed to enable family caregivers to assess, manage, and track BPSD.

**Methods:**

This RCT enrolled 57 dementia family caregivers from community and clinical settings in Ann Arbor, Michigan and Baltimore, Maryland. Participants were randomly assigned to immediate use of the WeCareAdvisor tool (WCA, *n* = 27) or a Waitlist control group (*n* = 30) that received the tool after a one-month waiting period. Outcomes for the caregiver and the person they were caring for were assessed at baseline (T0) and one-month followup for both the WCA (T1) and Waitlist control (T2) groups.

**Results:**

Caregiver mean age was 65.9 ± 14.0 years old. About half (49%) were spouses. Baseline characteristics were comparable between groups except for mean caregiver confidence which was higher in the control group (WCA 35.0 ± 10.0 vs. Waitlist control 39.7 ± 6.9, *p* = 0.04). There were no significant differences between the WCA and control groups in characteristics of the person with dementia. After their one-month of tool use (T1), WCA caregivers showed significant within group improvement in caregiver distress (− 6.08 ± 6.31 points, *t* = − 4.82, *p* < 0.0001) and behavioral frequency (− 3.60 ± 5.05, *t* = − 3.56, *p* = 0.002), severity (− 3.24 ± 3.87, *t* = − 4.19, *p* = 0.0003) and total behavioral score (− 6.80 ± 10.73, *t* = − 3.17, *p* = 004). In the same timeframe, Waitlist control caregivers showed a significant decrease in confidence (− 6.40 ± 10.30, *t* = − 3.40, *p* = 0.002). The WCA group showed greater improvement in distress compared to the Waitlist group (T0-T1; *t* = − 2.49, *p* = 0.02), which remained significant after adjusting for site and baseline distress. There were no significant between-group differences in caregiver confidence or other secondary outcomes. After their one month of tool use (T2), the Waitlist group also showed significant improvement in caregiver distress (− 3.72 ± 7.53, *t* = − 2.66, *p* = 0.013), stress (− 0.41 ± 1.02, *t* = − 2.19, *p* = 0.037), confidence (4.38 ± 5.17, *t* = 4.56, *p* < 0.0001), burden (− 2.76 ± 7.26, *t* = − 2.05, *p* = 0.05), negative communication (− 1.48 ± 2.96, *t* = − 2.70, *p* = 0.012) and behavioral frequency (− 1.86 ± 4.58, t = − 2.19, p = 0.037); distress remained significant after adjustment.

**Conclusions:**

In this pilot RCT, WCA use resulted in a significant decrease in caregiver distress. Future research will identify whether longer use of WCA can impact other caregiver and behavioral outcomes.

**Trial registration:**

Clinicaltrials.gov identifier NCT02420535 (Date of registry: 4/20/2015, prior to the start of the clinical trial).

## Background

Dementia currently affects over 5 million people in the US, and with the aging of the population is projected to affect 16 million people by 2050. While cognitive impairment is the clinical hallmark of dementia, non-cognitive behavioral and psychological symptoms (BPSD) are nearly universal (affecting 98% of individuals at some point in the illness course) and often overshadow cognitive symptoms in the disease course [1].

BPSD occur in dementias of all types and include depression, psychosis, psychomotor agitation, aggression, apathy, sleep disturbances, and inappropriate behaviors [2]. While BPSD can be thought of as the consequence of the neurodegeneration associated with dementia, they are triggered by multiple interacting factors internal and external to the person with dementia. These include patient (e.g. undiagnosed medical conditions and untreated pain), caregiver (e.g. ineffective communication style) and environmental (e.g. overstimulation or lack of activity/structure) factors [3]. Importantly, many of these triggers are modifiable.

There are 15 million family caregivers of people with dementia in the US. Clinicians depend on caregivers to assess and manage BPSD in the community including reporting on symptoms, carrying out recommendations, and evaluating the effect of treatment. As opposed to core cognitive symptoms, managing BPSD is one of the most challenging aspects of care, causing intense caregiver burden and upset [4]. Caregivers of individuals with BPSD are more distressed and depressed than those not managing behaviors [5]. There is emerging evidence that caregiver distress associated with BPSD is a more important predictor of institutionalization and inpatient and emergency department use than the frequency and severity of the BPSD themselves [6–9]. Thus, it is not surprising that BPSD are associated with a multitude of negative outcomes such as hospital stays, injuries, caregiver stress and depression, reduced caregiver employment income, earlier nursing home placement as well as higher patient mortality [3]. BPSD are also costly, with BPSD management accounting for 30% of the cost of caring for community-dwelling people living with dementia (PLWD) [10]. Recent research suggests that managing BPSD can lower costs of dementia care for families.

Unfortunately, few treatment options are currently available to family caregivers for BPSD. Typically, if a caregiver expresses concern about a BPSD to a physician, a psychiatric medication is prescribed to control the symptom. However, this is an ineffective and potentially dangerous strategy for several reasons: 1) the risk/benefit profiles of psychiatric medications for dementia are poor [11, 12]; and 2) BPSD are a “moving target” with different symptoms appearing over time and caregivers often managing multiple BPSD simultaneously. Unpredictability and complexity make a simple “magic bullet” medication solution impossible [13].

In contrast, nonpharmacologic strategies are recommended by multiple medical organizations and expert groups as the preferred first-line treatment approach to BPSD, except in emergency situations when behaviors could lead to imminent danger [14–16]. With an emerging evidence base, nonpharmacologic behavioral management strategies are increasingly recognized as a critical part of comprehensive, state of the art dementia care [15], with the common goals of symptom relief and reduction of caregiver distress. Currently, the best evidence base for non-pharmacologic approaches appears to rest with interventions for family caregivers [3] including Resources for Enhancing Alzheimer’s Caregiver Health (REACH II) [17], the Tailored Activity Program (TAP) [18] and the Care of Persons with Dementia in their Environments (COPE) [19], and the Advancing Caregiver Training (ACT) study [20]. In this type of approach, problem solving with a family caregiver to identify triggers or modifiable underlying causes of BPSD is combined with the use of selected non-pharmacologic strategies.

However, such approaches have largely not been translated to real-world care and clinical settings likely because they are “hands-on” staff- and training-intensive interventions. Such approaches have effectiveness, but require training and are time-consuming. Therefore, it is not surprising that: 1) few have been translated into a widely deliverable and sustainable approach, and 2) family caregivers continue to be underserved or receive services that are not evidence-based. Further, as families need assistance managing BPSD in real time, technologies and online platforms may be better able to deliver ‘on demand’ support [21]. While a number of prior studies have incorporated technology components [22–25], we know of no web-based, easy to use, comprehensive interactive tools to help family caregivers manage BPSD and track their modifiable underlying causes such as pain, infection, communication problems or environmental overstimulation including which strategies were effective.

We sought to overcome this research-practice gap by creating a web-based tool for family caregivers that would guide them through a clinical reasoning process to identify, monitor and manage behaviors while simultaneously addressing their motivation, self-efficacy and problem-solving skills. This approach has previously been applied to the management of complex health conditions such as cancer and asthma, or to assist with smoking cessation and weight management [26, 27].

The bedrock of the tool is the “DICE” approach to BPSD that was developed from a US national multidisciplinary expert consensus panel [28]. DICE™ (hereafter abbreviated as DICE) is an algorithmic, evidence-based approach comprising four steps: DESCRIBE: describe the behavior to derive an accurate characterization and the context in which it occurs; INVESTIGATE: examine, exclude and identify possible underlying causes of the behavior; CREATE: create and implement a treatment plan for the behavior; and EVALUATE: assess what parts of the treatment plan were attempted and effective. Within the DICE approach caregiver (expectations, caregiver stress/depression, etc), PLWD (medical conditions, functional status, etc); and environmental (overstimulation, lack of routines, etc) contributions to BPSD are evaluated and addressed.

This project had three phases. In the first, we involved end-users (e.g. family caregivers) in the design of a web-based tool incorporating DICE that would: 1) be easy to use; 2) be tailored to the PLWD and caregiver’s specific behavioral concerns, environment and personal characteristics; 3) teach new transferrable skills to the end-users; 4) provide an alternative to the risks and limited efficacy associated with medication treatment; and 5) could be used throughout the disease course. In the second phase, we engaged in iterative cycles of programming, testing and refinement to translate evidence-based treatment and knowledge into a usable tool (called the WeCareAdvisor). These two phases are described in detail in other manuscripts [29, 30].

In the third phase, we assessed the WeCareAdvisor tool (WCA) with family caregivers in a pilot randomized controlled trial (RCT) to evaluate its one-month effect on caregiver distress and caregiver confidence as compared to a waitlist control group. A one-month time frame for tool use was selected as appropriate for this pilot/proof of concept study. Secondary outcomes included caregiver stress, depression, burden, negative communication and relationship closeness, and PLWD behavioral frequency and severity. This paper reports the outcomes of the RCT including effects following one-month of tool use between an immediate treatment group (WCA) and a wait-list control group and then within group differences for the WCA and Waitlist control groups following their one month tool use.

## Methods

A two-site randomized controlled trial was conducted with one-month intervention and follow-up after baseline assessment targeting family caregivers of people with dementia. The design of the trial is detailed in a prior manuscript [30]. All three phases or the project were approved in 2012 by Institutional Review Boards of University of Michigan and Johns Hopkins University. This study adhered to CONSORT guidelines.

### Recruitment and eligibility

**Fig. 1:**
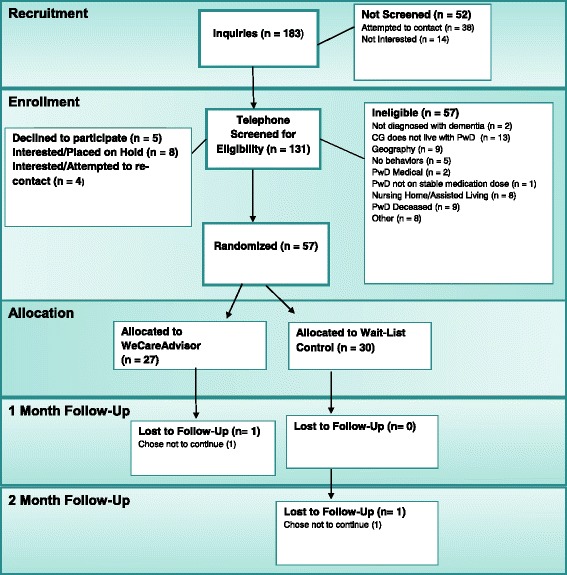
Consort Flow Diagram

Participants were consecutively recruited beginning in May 2015 through September 2016. They were recruited from community and outpatient clinical sites in Ann Arbor, Michigan and Baltimore, Maryland by several methods: provider or staff referral; on-site by research staff; caregiver response to flyers in a participating site; or participation in a previous trial. Interested caregivers were screened for eligibility by telephone. Inclusion criteria included: primary caregiver for a PLWD (clinical diagnosis or MMSE< 24); residing with the PLWD or close by; managing ≥ 1 behavioral symptom; English speaking; and familiarity with technology (computer, tablet or smartphone). Exclusion criteria included: caregiver sensory impairment; and for the person with dementia: imminent institutional placement, terminal illness, active suicide risk, or not on a stable dose of psychotropic for at least 60 days. Figure 1 depicts recruitment and study flow.

### Procedures

Eligible caregivers were next scheduled for a baseline in-home interview. Written consent from the caregiver and the PLWD were obtained at that time, including the fact that subjects could withdraw from the study at any point in time. If the PLWD was unable to provide informed consent, then proxy consent from the family caregiver (and assent from the person with dementia) was obtained. At the conclusion of the baseline interview, the interviewer opened a randomization envelope determining group assignment. Those randomized to the Waitlist control group (1-month wait) were informed of their assignment and an appointment in one-month was scheduled. Those assigned to receive the WeCareAdvisor immediately (intervention) received: 1) an iPad with the WeCareAdvisor website link; 2) optional email account setup (if no prior email access); and 3) approximately 15-min instruction in use of the tool.

Two to three days after the baseline visit and thereafter weekly during the one-month of the intervention, participants received a “check-in” phone call from a study team member. The purpose of this call was to trouble shoot problems with the tool and encourage tool use. These calls were not of a clinical nature; staff used a guiding script for the purpose of this call in order to anchor it around the tool, and limited the length of calls to a maximum of 20 min.

One month after baseline, an in-home follow-up interview was completed with both intervention and control participants. For intervention participants, after outcome assessment, the interviewer collected the iPad and their study participation was concluded. For Waitlist controls, identical procedures were followed as for the intervention group at baseline, and this group began their one month of tool use and was reassessed one month later.

### Randomization

A randomization table was created by the University of Michigan statistician in Stata, stratified by study site and using random blocks of sizes 4,6 or 8, and provided to a project manager at each site who was not involved in assessment. The project manager created sequentially numbered sealed envelopes containing group assignment. Each consenting caregiver received the envelope in the order in which they were enrolled. The project manager had access to the spreadsheet that indicated the randomization order but the research assistants who performed baseline and outcome assessments were masked as to randomized condition.

### Baseline assessments

Comprehensive assessments were performed to collect data from caregivers and people with dementia on several domains including: 1) demographics; and 2) physical and mental health related characteristics; 3) and technology experience (caregiver only). Primary and secondary outcomes are below.

### Primary and secondary outcomes

The primary study aim was to evaluate the extent to which WCA impacted caregiver distress (measured by the total of the distress items from a revised version of the NPI-Q; in the revised version, we separated agitation and aggression and added frequency for a total of 14 items, score range 0–70) and confidence in using activities to manage BPSD (five item scale, score range 0–50) after one month of use. Caregiver distress was selected as the primary outcome based on emerging evidence that it may be a more important predictor of institutionalization and inpatient and emergency department use than the frequency and severity of the BPSD themselves [6–9]. We expected that after one month of WCA use, the intervention group would report less distress and greater confidence than those in the Waitlist control group. We also expected that the Waitlist group would experience similar benefits following their one month of tool use.

Secondarily, we evaluated change in caregiver stress (five-point likert scale ranging from 1 to 5), depression (CES-D, 20 items, score range 0–60), burden (Zarit burden scale,12-item, score range 12–60), negative communication (Negative Communication Scale, 6 items, score range 6–30) and relationship closeness (Relationship Closeness Scale, 6-item, score range 6–24) and PLWD behavioral frequency, severity, and total behavioral score (NPI-Q revised version, separating agitation and aggression and adding frequency as in the NPI-C). Secondary measures were chosen based upon those that are both meaningful in clinical practice as well as appear to be impacted by family caregiver interventions in prior trials [18–20]. For a full description of measures, please see the WeCareAdvisor protocol paper [30].

### Intervention/components of the WeCareAdvisor tool

For a full description of the tool, please refer to the prior paper describing its development [29]. Briefly, the tool contains three main components.

- The first is a guided DICE approach where a peer navigator (tailored to the age, race and gender of the caregiver) leads the caregiver through the approach; this occurs through the caregiver answering questions related to symptom context (who, what, when, where) and possible medical/pain issues including delirium. Based upon these answers, an algorithm selects from over 900 evidence-based strategies to create a WeCareAdvisor “prescription”. Caregivers are instructed to try the strategies for one week and then evaluate how the strategies work for them; if the strategies are helpful, they are encouraged to keep using them, if the strategies have not been helpful, caregivers are encouraged to conduct another DICE session to get a new set of strategies. During orientation, caregivers conducted their first DICE session on the most problematic behavior they were experiencing, and during their one-month trial were encouraged to create as many sessions as they feel would be helpful to them.
- The second component is the Caregiver Survival Guide which is a compendium of information for dementia caregivers (e.g. “what is dementia”, “keeping the person with dementia healthy”) located in one place for “one stop shopping”.
- The third component is a daily messaging feature that provides an encouraging daily communication to caregivers for support and motivation.

### Analytic approach

Data management and analyses were coordinated at the University of Michigan for both sites. For all outcome measures, summary descriptive statistics were calculated at baseline and one month, for change scores from baseline to one month by study group, and by site for one month to two months (for the Waitlist control group). WCA-associated change was estimated in a regression model with change-scores from baseline to one month as the dependent variable, and an indicator for the intervention group as the primary independent variable. The model adjusted for baseline values of the outcome variable (e.g. for change in confidence, adjustment for baseline confidence) and site (Ann Arbor vs. Baltimore). The parameter estimate of the intervention group indicator allowed for testing of the WCA effect on distress (or confidence) and estimated expected changes in scores associated with the tool, adjusting for baseline values. Due to presence of two participants with missing follow-up data, to obtain unbiased estimates, we also used a mixed model and obtained an estimate associated with the tool by fitting a multilevel model with both baseline and follow-up data as dependent variables. The model included random intercepts for participants, site, time, WeCare group, and time by WeCare group interaction term. Because the Waitlist controls received the tool after a one-month delay, their change data from one to two months was used to estimate additional effects in this group. We also compared intervention to waitlist changes in caregiver stress, depression, burden, negative communication, and relationship closeness as well as in the frequency, severity and total score for behavioral symptoms in PLWD.

### Power consideration

Although this was a proof of concept and feasibility Phase II trial, our proposed sample size of 30 dyads in each study group (*n* = 60 total) was calculated to afford 80% power, based on a two-group 0.05 significance level test, to detect a standardized effect size of 0.725, and the margin of error for the effect size estimate with 95% confidence calculated to be ± 0.51 standard deviation.

## Results

Of 131 potential participants screened across the two sites, 74 caregivers were eligible. Of eligible caregivers, 57 (77.02%) were willing to participate. A total of 55 (96.5%) completed followup. The mean age of caregivers was 65.9 ± 14.0 years old and the majority (75%) were female. Most (84%) had more than a high school education and the majority (74%) were married and white (63%). Almost half (49%) were spouses. Caregivers had a mean of 4.1 ± 3.1 medical and 0.4 ± 0.6 mental health conditions. Regarding PLWD, their mean age was 80.4 ± 10.2 years old, most were female (63%), married (58%), and white (65%). The mean MMSE was 16.5 ± 8.3, the mean number of NPI behaviors 7.3 ± 3.1 and the mean level of functional dependence 9.9 ± 4.3.

Table 1 shows baseline (T0) demographic characteristics for caregivers and the PLWD in the WCA and Waitlist control groups. A Wald χ^2^ test from a logistic regression controlling for site indicated there were no significant demographic differences between the two study groups. Table 2 lists other characteristics for caregivers and the PLWD showing no significant differences between the groups with the exception of caregiver confidence in using activities to manage behaviors that was significantly higher at baseline in the Waitlist group, based on a Wald χ^2^ test from a logistic regression controlling for site.

**Table 1:** Basic Demographic Characteristics (*N* = 57)

	WeCare^g^ (*N* = 27)		Waitlist^g^ (*N* = 30)		
					*p*-value^b^
Caregiver	N	(%)	N	(%)	
Age, Mean ± S.D.	65.5 ± 11.8		66.2 ± 15.9		0.882
Male	8	[29]	6	[19]	0.405
Education^a^					
< =High School /GED	4	[15]	5		0.858
> High School	23	(85)	25	(83)	
Marital Status^a^					
Single	10	(37)	5		0.087
Married	17	(63)	25	(83)	
Race^a^					
White	17	(63)	19	(63)	0.788
African American	8	[29]	10	(33)	
Other	2	[7]	1	[3]	
Person with Dementia is Spouse^a^	13	(48)	15	(50)	0.870
No. Physical Chronic Conditions (0–24)^c^	4.2 ± 3.4		4.1 ± 2.9		0.906
No. Mental health diagnoses (0–5)^d^	0.3 ± 0.6		0.5 ± 0.6		0.310
Person with Dementia					
Age, Mean ± S.D.	82.3 ± 9.3		78.6 ± 10.8		0.102
Male	8	[29]	13	(43)	0.286
Marital Status^a^					
Single	13	(48)	11	(37)	0.374
Married	14	(52)	19	(63)	
Race^a^					
White	18	(67)	19	(63)	0.706
African American	7	[25]	10	(33)	
Other	2	[7]	1	[3]	
MMSE (0–30), Mean ± S.D.	15.0 ± 8.5		18.0 ± 8.0		0.091
No. of Behaviors^e^ (0–14), Mean ± S.D.	7.6 ± 3.0		7.0 ± 3.3		0.462
Functional Dependence^f^ (0–15), Mean ± S.D.	10.4 ± 4.5		9.5 ± 4.1		0.378

^a^Categories are collapsed

^b^For between study arm difference controlling for site

^c^Count out of 24 systemic illnesses and conditions

^d^Count out of 5 mental health diagnoses

^e^Number of presence of 14 NPI symptoms

^f^Number of assistance needed in 15 daily function items

^g^One subject dropped in follow-up

**Table 2:** Baseline Characteristics of Caregiver and Person with Dementia (N = 57)

	WeCare (N = 27)	Waitlist (*N* = 30)	*p*-value^a^
Caregiver	N	N	
Confidence in Using Activities (5 items; 0–50)^b^	35.0 ± 10.0	39.7 ± 6.9	0.044
Technology Experience^c^	9.3 + 1.0	8.8 + 1.4	0.146
Caregiver Distress (14 items; 0–70)^d^	18.0 ± 10.7	15.4 ± 10.7	0.372
Caregiver Stress (1–5)^d^	3.0 ± 1.2	2.8 ± 1.1	0.586
CES-D for Depression (20 items; 0–60)^d^	12.6 ± 8.9	12.6 ± 7.5	0.984
Burden (12 items; 12–60)^c^	31.9 ± 7.3	31.2 ± 8.2	0.743
Negative Communication (6 items; 6–30)^d^	11.5 ± 3.4	11.8 ± 3.5	0.701
Relationship Closeness (6 items; 6–24)^d^	17.3 ± 4.7	17.6 ± 4.4	0.867
Caregiver Readiness (17 items; 17–68)^d^	59.0 ± 5.6	57.1 ± 6.8	0.253
Person with Dementia			
Overall Physical Health (1–5)^e^	3.2 ± 1.2	3.1 ± 1.2	0.729
No. of Health Assessment (of 25 Systemic Illnesses and Conditions; 0–25)	6.2 ± 3.0	6.0 ± 3.3	0.854
Behavior Frequency (14 items; 0–56)^f^	18.8 ± 8.8	15.4 ± 9.1	0.164
Behavior Severity (14 items; 0–42)^f^	12.8 ± 7.7	10.4 ± 6.4	0.224

^a^For between study arm difference controlling for site

^b^Can range 0–50; 5 items, each 0–10. Higher scores correspond to greater confidence

^c^Can range from 0 to 10; use of computer, internet, cell phone, microwave oven, copier, ATM, self-checkout at grocery store, audio books, digital camera, and programmable devices (e.g. coffee maker, thermostat)

^d^Higher scores correspond to greater distress, stress, depressive symptoms, burden, negative communication,

closeness, or readiness, respectively

^e^Excellent [5], Very Good [4], Good [3], Fair [2], Poor [1]

^f^Higher scores correspond to greater frequency or severity, respectively

We examined demographic and other characteristics by study site using t-tests for continuous variables and χ^2^ tests for categorical variables (Ann Arbor vs. Baltimore) where several significant differences were noted: caregiver age (mean of 62.0 ± 14.3 in Ann Arbor vs. 69.4 ± 12.9 in Baltimore, *p* = 0.04), PLWD age (Ann Arbor mean 75.1 ± 10.2 vs. Baltimore mean 85.1 ± 7.5, *p* < 0.001), MMSE (Ann Arbor mean 19.7 ± 7.6 vs. Baltimore mean 14.1 ± 8.2, *p* = 0.02) and functional dependence (Ann Arbor mean 8.0 ± 4.4 vs. Baltimore mean 11.6 ± 3.4, *p* = 0.001).

### Tool use

Table 3 shows the heterogeneity of behaviors that caregivers used the WCA for during the trial. The most common behavior caregivers created DICE sessions for was agitation (17.5%) followed by toileting (12.3%), aggression (10.5%), and anxiety (10.5%). DICE sessions were created for all 10 other behaviors but in fewer than 10% of caregivers each.

**Table 3:** Behaviors Targeted by Caregivers in DICE Sessions

Behavior targeted in DICE sessions	Number of caregivers	PERCENT
Agitation	10	17.5
Toileting	7	12.3
Aggression	6	10.5
Anxiety	6	10.5
Apathy	5	8.8
Appetite and Eating	5	8.8
Motor Disturbance	5	8.8
Delusions	3	5.3
Irritability	3	5.3
Nighttime Behaviors	3	5.3
Depression	2	3.5
Disinhibition	1	1.8
Hallucinations	1	1.8

### One-month Followup outcomes

Table 4 presents the results of unadjusted followup outcomes.

**Table 4:** Change from Baseline to Follow-up in Caregiver and Person with Dementia Measures

	Change from Baseline to Month 1 (value at month 1- value at baseline)								Change from Month 1 to Month 2 (value at month 2- value at month 1)		
	WeCare (n=26^a^)			Waitlist (N = 30)			Test for group difference in change (WeCare-Waitlist)		Waitlist (N=29^b^)		
Caregiver	Mean Change± S.D.	t-value	p-value	Mean Change ± S.D.	t-value	*p*-value	t-value	*p*_value	Mean Change ± S.D.	t-value	*p*-value
Confidence in Using Activities (5 items; 0–50)^d^	1.00 ± 11.44	0.45	0.660	−6.40 ± 10.30	−3.40	0.002	2.55	0.014	4.38 ± 5.17	4.56	<.0001
Caregiver Distress (14 items; 0–70)^d^	− 6.08 ± 6.31	−4.82	<.0001	−1.80 ± 6.36	−1.55	0.132	− 2.49	0.016	−3.72 ± 7.53	− 2.66	0.013
Caregiver Stress (1–5)^d^	0.12 ± 1.66	0.36	0.726	0.17 ± 0.99	0.93	0.362	−0.14	0.891	−0.41 ± 1.02	− 2.19	0.037
CES-D for Depression (20 items; 0–60)^d^	− 2.08 ± 5.87	−1.77	0.089	− 0.47 ± 7.13	− 0.36	0.723	−0.90	0.370	−0.55 ± 8.63	−0.34	0.733
Burden (12 items; 12–60)^d^	− 1.72 ± 6.91	−1.25	0.225	1.30 ± 6.38	1.12	0.274	−1.68	0.098	− 2.76 ± 7.26	−2.05	0.050
Negative Communication (6 items; 6–30)^d^	− 1.04 ± 3.56	−1.46	0.158	0.77 ± 3.62	1.16	0.255	−1.86	0.069	−1.48 ± 2.96	−2.70	0.012
Relationship Closeness (6 items; 6–24)^d^	0.36 ± 2.33	0.77	0.446	−0.37 ± 4.31	− 0.47	0.645	0.79	0.431	1.07 ± 3.30	1.72	0.097
Person with Dementia											
No. of Behaviors^e^ (0–14)	−1.62 ± 1.77	− 4.66	<.0001	−0.93 ± 2.15	−2.38	0.024	−1.28	0.204	−0.38 ± 1.82	− 1.12	0.272
Behavior Frequency (14 items; 0–56)^f^	− 3.60 ± 5.05	−3.56	0.002	−1.90 ± 5.63	−1.85	0.075	−1.17	0.248	−1.86 ± 4.58	− 2.19	0.037
Behavior Severity (14 items; 0–42)^f^	− 3.24 ± 3.87	−4.19	0.0003	− 1.47 ± 4.04	−1.99	0.056	−1.65	0.104	−1.45 ± 4.50	−1.73	0.094
NPI Score^f^ (frequency x severity)	−6.80 ± 10.73	−3.17	0.004	−3.20 ± 10.67	−1.64	0.111	−1.24	0.220	−5.00 ± 14.34	− 1.88	0.071

^a^One subject dropped in Month-1 follow-up

^b^One subject dropped in Month-2 follow-up

^c^Can range 0–50; 5 items, each 0–10. Higher scores correspond to greater confidence

^d^Higher scores correspond to greater distress, stress, symptoms, burden, negative communication, or closeness,respectively

^e^Number of presence of 14 NPI symptoms

^f^Higher scores correspond to greater frequency, severity, or score, respectively

#### Within group outcomes baseline to one month (T0-T1)

Based on a t-test, within the group that immediately received the WeCareAdvisor tool, distress declined significantly after their one month of tool use (6.08 ± 6.31 points, *t* = − 4.82, *p* < 0.0001); confidence increased after tool use, but this increase was not significant (1.00 ± 11.44 points, *t* = 0.45, *p* = 0.66). The frequency of behaviors (− 3.60 ± 5.05 points, *t* = − 3.56, *p* = 0.002), severity of behaviors (− 3.24 ± 3.87 points, *t* = − 4.19, *p* = 0.0003) and NPI score (− 6.80 ± 10.73 points; *t* = − 3.17, *p* = 0.004) were all significantly decreased in the WCA group after one month of tool use. Distress (*t* = − 4.51, *p* < 0.001), behavioral frequency (*t* = − 3.31, *p* = 0.0014), severity (*t* = − 3.76, p = 0.0003) and NPI score (*t* = − 2.53, *p* = 0.0132) all remained significant after adjustment for site. In the same timeframe, Waitlist control caregivers showed a significant worsening (decrease) in confidence (− 6.40 ± 10.30, *t* = − 3.40, p = 0.002).

#### Between group comparisons

##### Caregiver distress

At one-month follow-up (T1), a χ^2^ test showed the WCA group had a higher percentage of caregivers with improvement in distress (number of caregivers who had any improvement in distress from T0 to T1 divided by the total number of caregivers in the group) over the Waitlist control group (73.1% vs. 46.7%, χ^2^ = 4.01, df = 1, *p* = 0.04). Unadjusted analyses using a t-test showed that the WCA group had a greater decline in distress compared to the Waitlist control group (*t* = − 2.49, *p* = 0.02). Results remained significant after adjusting for site and baseline distress in a regression model (beta = − 3.71 p = 0.02) and in a mixed model (beta = − 4.45, p = 0.02).

##### Caregiver confidence

At one-month follow-up (T1), the WCA group had a higher percentage of caregivers with improvement in confidence over the Waitlist control group (42.3% vs. 26.7%), however this difference was not significant based on a χ^2^ test (χ^2^ = 1.52, df = 1, *p* = 0.22). In unadjusted analyses, the WCA group had a significantly greater change in confidence than the Waitlist control group (t-2.55, *p* = 0.01); note that this difference did not indicate significant improvement in confidence in the WCA group, but rather worsening confidence in the Waitlist group. The difference was not significant in a regression model after adjusting for site and baseline level of confidence (beta = 4.48, *p* = 0.09), however, remained significant in mixed model (beta = 7.52, *p* = .004).

##### Caregiver stress, depression, burden, negative communication, relationship closeness

No differences between study groups in these outcomes were found in unadjusted and adjusted analyses using both regression and mixed model.

##### Person living with dementia behavioral outcomes

There were no significant differences in behavioral frequency, severity or overall NPI score (frequency by severity) between the WCA and Waitlist groups at one-month followup based on t-tests and in regression and mixed models.

#### Waitlist control one-month within group outcomes (T1-T2)

A t-test indicated that the Waitlist control group showed significant improvement in both primary outcomes after their one month (T1 to T2) of tool use: distress significantly decreased (3.72 ± 7.53 points, *t* = − 2.66, *p* = 0.013) and confidence significantly improved (4.38 ± 5.17 points, *t* = 4.56, *p* < 0.0001). This group also showed improvement in stress (− 0.41 ± 1.02 points, *t* = − 2.19, *p* = 0.04), caregiver burden (− 2.76 ± 7.26 points, *t* = − 2.05, *p* = 0.05), and negative communication (− 1.48 ± 2.96 points, *t* = − 2.70, *p* = 0.012). PLWD in the Waitlist group also showed a significant reduction in frequency of behaviors (− 1.86 ± 4.58 points, t = − 2.19, *p* = 0.037) and trend-level reductions for severity of behaviors (− 1.45 ± 4.50 points, *t* = − 1.73, *p* = 0.09) and overall NPI score (− 5.00 ± 14.34 points, *t* = − 1.88, *p* = 0.07).

The significant decline in distress between T1 and T2 for the Waitlist control group remained significant after adjustment for site in a mixed model (beta = − 3.83, *p* = 0.004) as did the significant improvement in confidence (beta = 4.67, *p* = 0.009). However, stress (beta = − 0.43, *p* = 0.06) and the decrease in frequency of behaviors was not significant after adjustment for site in a mixed model (beta = − 1.90, *p* = 0.075).

**Fig. 2:**
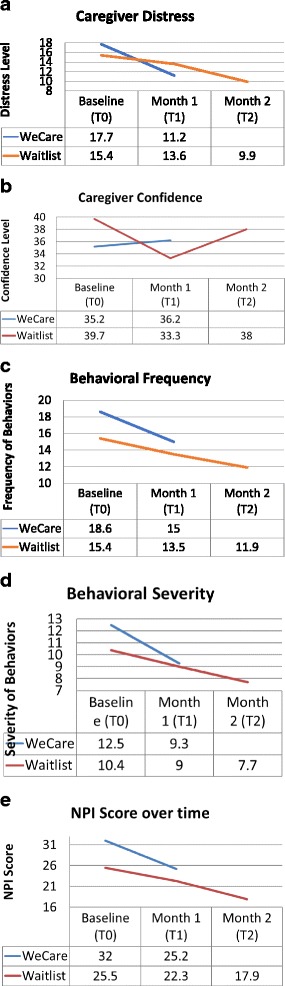
Change in Caregiver Distress and Confidence and PLWD Behavioral Frequency, Severity and NPI Total Score Over Time

Figure 2a shows the pattern for caregiver distress for the two study groups over the trial. Figure 2b shows the pattern for caregiver confidence for the two study groups over the trial. Figure 2c, d and e show behavioral frequency, severity and overall NPI score for the two groups across the trial.

## Discussion

In this pilot RCT, we tested a novel web-based tool to help caregivers manage BPSD on caregiver distress and confidence over a one-month period. Use of the WCA was associated with significant declines in caregiver distress in the primary comparison of the intervention group vs. the waitlist control group, as well as after the waitlist control group completed one month use of the tool. The impact on caregiver confidence was less clear, with unadjusted differences reflecting worsening of confidence in the control group. Interestingly, however, once the control group completed their use of the tool, they showed improvement in confidence that remained after adjustment. While PLWD behavioral frequency, severity, and overall NPI score were null in the primary comparison of the WCA vs. Waitlist group, within group comparisons showed improvement with use of the tool (for the WCA group, frequency, severity and total behavioral scores; for the Waitlist group, trend-level improvement in behavioral frequency).

The impact of the WeCareAdvisor on caregiver distress suggests that it could have a role in caregiver management of BPSD. Emerging evidence suggests that the impact of behaviors on caregivers, manifested as distress, is an important predictor of negative outcomes. Philp et al. found that among 114 community-dwelling Scottish people with dementia supported by family carers followed for 2 years, caregiver perceptions of a behavior as problematic rather than severity of a behavior independently predicted institutionalization [9]. In a second study of 119 Dutch PLWD and their caregivers followed over five years, caregiver distress (measured using the NPI as in the present study) itself was a significant predictor of nursing home placement while behavior itself was not [8]. The authors noted that their findings could indicate that a caregiver’s emotional reaction to a particular behavior was more important than the behavior per se in the decision to admit the person with dementia to a nursing home. A meta-analysis of BPSD and outcomes concluded that caregiver factors are more important than care-recipient factors in predicting institutionalization [6]. A recent analysis using retrospective, nationally representative data of participants with dementia from the Aging, Demographics and Memory study found that high levels of caregiver distress (rather than the BPSD themselves) were significantly associated with emergency department visits, hospitalizations, and costs [7]. Cumulatively, this evidence suggests that identifying and supporting caregivers in distress is a worthy target in reducing the negative outcomes associated with BPSD.

Notably, outcomes related to caregiver confidence were less clear. In part, this may be related to the higher baseline level of mean confidence in the control group. Another factor may be the confidence measure used in this trial (that assesses confidence in using activities to manage BPSD). This measure has been used in prior studies of caregiver interventions related to the use of tailored activities [18, 31]. While WeCareAdvisor applies a number of strategies involving use of activity to manage some symptoms (e.g., agitated outbursts), activity may be less relevant for other problems (e.g. for certain toileting problems). While agitation was the most common problem behavior for which caregivers created DICE sessions, the second most common was toileting; in these cases, confidence using activities is an imperfect measure. Thus, it is possible that this measure was not sensitive to the broad array of behaviors that family caregivers in this study were managing.

Similarly, while the findings for behavior change trended in a promising direction, there was no significant impact of WCA use on severity or frequency of BPSD as compared to the control group in the primary comparison. There may be several reasons for this. First, we did not select for any behaviors (e.g. agitation) like other trials and therefore heterogeneity (with behaviors ranging from agitation to apathy to eating issues, etc) may have impacted results. Second, one month may not be enough time to impact behavioral outcomes; notably most drug trials in BPSD are 8–10 weeks. These aspects of our trial may have impacted the ability to effect overall stress level, depression, caregiver burden, negative communications or relationship closeness.

Strengths of the study include development of a low cost, easy to use, on-demand web-based intervention for caregivers to address BPSD, developed with extensive end-user feedback. Another strength is that the tool is based upon the DICE Approach which emphasizes the importance of looking for underlying causes (e.g. pain, UTI) with symptoms like agitation. A final strength is that this was a controlled trial with dyadic outcomes which are critical in the evaluation of impact on BPSD. Weaknesses include a control group with limited attention and support (e.g. the intervention group received about 1 h more of staff contact via weekly check-in calls about tool use over 1 month) and positive outcomes in the primary comparison limited to caregiver distress. While this small pilot RCT demonstrates feasibility and a promising impact on caregiver distress, the size of and limitations inherent in this study limit generalizability. A future study will include a longer (three month) trial with a larger more diverse sample including attention to whether the weekly check-in calls are necessary or whether email prompts are sufficient. A long-term care version of the tool is also currently being tested in Veterans Administration Community Living Center (nursing home) facilities.

## Conclusions

In our pilot RCT, use of the WCA resulted in significant decrease in caregiver distress. Prior research has shown that caregiver distress is an important predictor of negative BPSD outcomes including ED visits, hospitalizations and nursing home placement. Future research will identify whether longer use of WCA can significantly impact other caregiver and behavioral outcomes.

## Abbreviations

BPSD: Behavioral and psychological symptoms of dementia
CES-D: Center for Epidemiologic Studies Depression scale
DICE: Describe, Investigate, Create, Evaluate
NPI: Neuropsychiatric Inventory
PLWD: Person living with dementia
RCT: Randomized controlled trial
T0: Baseline
T1: One-month followup
T2: One-month follow-up post tool for Waitlist control group
WCA: WeCareAdvisor
